# Tissue-specific metabolomic signatures for a *doublesex* model of reduced sexual dimorphism

**DOI:** 10.1098/rsos.250770

**Published:** 2025-07-09

**Authors:** Rene Coig, Benjamin Harrison, Richard Johnson, Michael J. MacCoss, Daniel Promislow

**Affiliations:** ^1^ Department of Laboratory Medicine and Pathology, University of Washington School of Medicine, Seattle, WA, USA; ^2^ Department of Genome Sciences, University of Washington, Seattle, WA, USA; ^3^ Jean Mayer USDA Human Nutrition Research Center on Aging, Tufts University, Boston, MA, USA

**Keywords:** *Drosophila melanogaster*, *Doublesex*, sexual dimorphism, sex differences, metabolomics

## Abstract

Sex has a major effect on the metabolome. However, we do not yet understand the degree to which differences in metabolism are associated with anatomical dimorphism and modulated by sex-specific tissues. In the fruit fly, *Drosophila melanogaster*, knocking out *doublesex* (*dsx*) gives rise to adults with intermediate sex characteristics. Here, we sought to determine the degree to which this key node in sexual development leads to sex differences (SD) in the metabolome. We measured 91 metabolites across three tissues, comparing sex-dimorphic flies with those of reduced dimorphism: *dsx* null flies. The abundance of 51% of metabolites (46/91) differed between wildtype XX and XY flies in at least one tissue. However, in *dsx* flies, we only observed a sex difference in kynurenate, suggesting that *dsx* plays a major role in SD in fly metabolism. Kynurenate was consistently higher in XX flies in both *dsx* flies and controls. We also observed tissue-specific effects in *dsx* flies. Sex dimorphism manifests in part through dimorphic growth of organs, and we find that dimorphic metabolites across the fly enriched the growth-related branched-chain amino acid and mammalian target of rapamycin pathways. Our findings demonstrate that sex dimorphism is accompanied by substantial effects on the metabolome throughout the body.

## 1. Introduction

Sex differences (SD) in biological processes are pervasive and have far-reaching implications for health and disease [1–3]. In *Drosophila*, these differences extend beyond reproductive functions and include many aspects of cellular metabolism [4–8]. In SD research, a distinction can be made between sexual dimorphism (the categorical, anatomical difference in morphology between the sexes) and SD (the quantitative, bimodal distribution of quantitative traits between the sexes) [9]. Currently, there is a significant gap in our understanding of how variation in anatomical sexual dimorphism impacts quantitative SD in metabolism.

The metabolome consists of the entire spectrum of small-molecule metabolites found within a biological sample [10]. These metabolites, such as lipids, amino acids, nucleotides and other small molecules, offer a snapshot of an organism’s physiological state [11,12]. Metabolome profiles can vary significantly between sexes [13–15] and genetic factors influence the *Drosophila* metabolome [16–19]. Thus, genes that lead to sexually dimorphic anatomy might also lead to SD in metabolite levels. Furthermore, variants in sex development genes that increase or decrease the degree of anatomical dimorphism may lead to similar increases or decreases in the effects of sex observed in the metabolome.

Different tissues in an organism perform specialized functions, and their metabolic needs and activities are tailored to support these functions. For example, muscle tissue requires energy for contraction and movement, while the fat body in a fly is involved in detoxification and glucose metabolism. Sex-specific tissues such as ovaries or testes may have unique metabolic demands to perform their specialized functions related to reproduction. Furthermore, communication between reproductive organs and other tissues may influence the overall metabolic landscape of the body. For example, 20-hydroxyecdysone circulating in the hemolymph can influence *Drosophila* oocyte development [20]. Currently, there is a gap in our understanding of how the presence, absence or variation in the function of sex-specific tissues such as ovaries or testes may affect SD in the fly metabolome.

This study aims to address these gaps by measuring the tissue-specific metabolome in a *Drosophila* model of reduced sexual dimorphism. The *Drosophila* sex development pathway is well characterized [21,22], providing an excellent system for genetic manipulation of sexual dimorphism. In *Drosophila*, the *doublesex* (*dsx*) gene acts in a global alternative splicing cascade, which results in female- and male-specific protein isoforms of *dsx* (DSX^F^ and DSX^M^) that are essential for determining the sexual fate of cells throughout the organism [23–25]. Knockout of *dsx* disrupts this cascade, resulting in incomplete development of gonads and genitals [26]. *Dsx* is also known to function as a tissue-specific regulator during development [27–29]. Throughout adulthood, *dsx* gene products continue to be expressed in the gonads [30] as well as the head [31], particularly in the central nervous system [32,33], and in the fat body [27]. This makes *dsx* an exceptionally powerful gene for unravelling the tissue-specific metabolic consequences of sexual dimorphism in *Drosophila*.

In this study, we use liquid chromatography-mass spectrometry (LC-MS) targeted metabolomic analysis to compare metabolome profiles of distinctly sex-dimorphic wildtype flies with those of reduced sexual dimorphism *dsx* null flies across head, thorax and abdominal tissues. We hypothesized that reduced dimorphism in *dsx* null flies would be associated with a reduction in SD observed in the metabolome. The primary focus of this analysis is to determine the extent and composition of *dsx*-dependent SD. We refer to each group of XX and XY flies (wildtype or *dsx* null) as a sex difference group (SD group) and compare the effect sizes of SD in the metabolome between these SD groups. Our findings reveal that SD in the metabolome are greatly reduced in the *dsx* null sexes as compared to wildtype. While *dsx* knockout led to a loss of significant SD in almost all metabolite features, the specific features that were significantly different between wildtype sexes varied among the three tissues. Our work focuses on a mutant with nearly indistinguishable anatomical SD, though these results highlight the importance of considering anatomical sexual dimorphism more generally as a modulator of SD in the metabolome.

## 2. Results

We used LC-MS to measure metabolite concentrations in head, thorax and abdomen tissues of four types of flies in a *w*^1118^ genetic background: XX-wildtype, XX-*dsx* null, XY-*dsx* null and XY-wildtype flies. We first report and compare SD observed in the metabolome for both SD groups. Next, we report the tissue-specificity of these SDs. Last, we report pathway analysis results for enrichment among metabolites with significant effects of sex.

### 2.1. Reduction in sexual dimorphism is accompanied by a reduction in global sex difference in the metabolome

The primary purpose of this study was to query the degree to which the sex determination pathway impacts SD in metabolism. We hypothesized that global SD in the metabolome of *dsx* null flies would be significantly reduced from that of wildtype flies. Principal component analysis (PCA) of all samples together strongly separated tissues from one another, with PC1 segregating abdomen samples from both head and thorax, and PC2 discriminating head from thorax (electronic supplementary material, figure S1). We thus analysed the data from each tissue separately.

**Figure 1.**
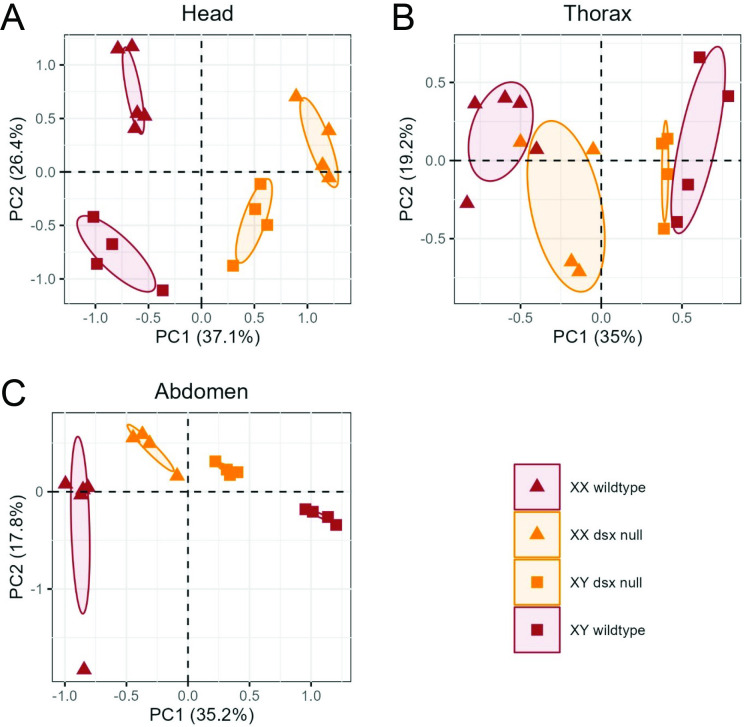
PCA of metabolome samples by tissue. PCA for (A) head tissue, (B) thorax tissue and (C) abdomen tissue. *dsx* null flies are coloured in orange, wildtype flies are coloured in brown. Triangles represent XX and squares represent XY fly samples.

Within each tissue, PCA separated all four genotypes from each other, with the *dsx* null metabolome profiles largely intermediate to those of wildtype flies in PC1 in the thorax and abdomen and in PC2 for head tissue (figure 1, electronic supplementary material, table S1A). However, for other PCs, the metabolome of *dsx* null samples fell outside the ranges of the wildtype (such as PC1 in head and PC2 in thorax), indicating that no single axis can distinguish the sexes in *Drosophila*. Notably, there was little overlap in the metabolomes of all four groups of flies regardless of tissue (figure 1).

**Figure 2.**
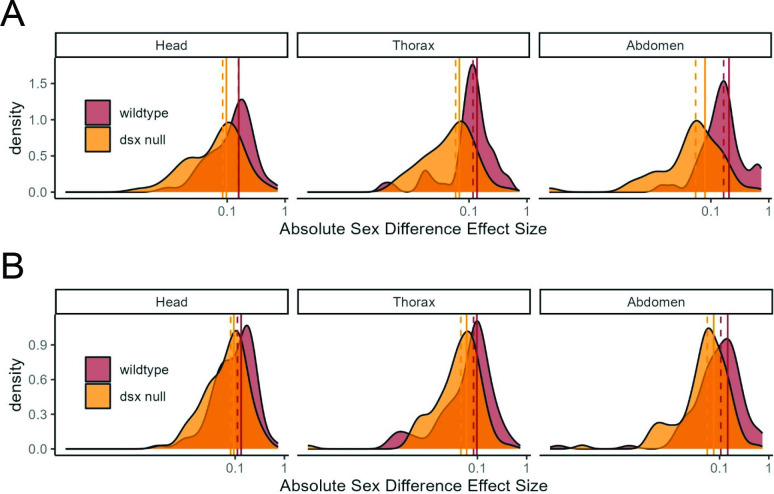
Density of sex effect sizes across metabolites in wildtype and *dsx* null sexes. (A) Density plot across SD metabolites, those metabolites with a significant effect of sex in wildtype flies (*n* = 46 metabolites). Sex effect sizes across metabolites are significantly different between wildtype and *dsx* null groups: head (*p* = 0.002), thorax (*p* = 3 × 10^-4^) and abdomen (*p* = 1 × 10^-6^). (B) Density plot across all metabolites (*n* = 91 metabolites). Sex effect sizes across metabolites are significantly different between wildtype and *dsx* null groups: head (*p* = 0.007), thorax (*p* = 0.02), abdomen (*p* = 6x10^-4^). Vertical lines mark the median (dashed) and mean (solid) effect size across metabolites. Effect sizes are plotted as absolute values on a log_10_ scale.

We next tested for SD in individual metabolites between SD groups. As expected, we observed strong SD in the metabolome of wildtype flies, with 46 metabolites (51%) showing significant SD at FDR < 5% in at least one tissue (table 1, electronic supplementary material, table S1B). We refer to these metabolites as ‘SD metabolites’. We found no indication of a sex bias in the directionality of SD metabolites with, for example, higher levels in XX as compared to XY (table 2). Comparisons of the magnitude of sex effect sizes among the SD metabolites in SD groups confirmed that SDs were significantly reduced in the *dsx* null head (*p* = 0.002), thorax (*p* = 3 × 10^-4^) and abdomen (*p* = 1 × 10^-6^) (figure 2A). Including all metabolites in the analysis, the reduction was less significant: head (*p* = 0.007), thorax (*p* = 0.02), abdomen (*p* = 6 × 10^-4^) (figure 2B).

**Table 1.** Summary of sex effect magnitude across metabolites by tissue.

		N metabolites FDR <5%	% metabolites FDR <5%	mean ES metabolites FDR <5%	mean ES all metabolites
	head	22	24%	0.16	0.13
wildtype	thorax	33	36%	0.14	0.10
	abdomen	24	26%	0.21	0.15
	head	1	1%	0.10	0.09
dsx null	thorax	1	1%	0.07	0.06
	abdomen	1	1%	0.08	0.08

Metabolites with a significant effect between sexes (referred to as SD metabolites) at FDR <5%. Effect size (ES) is calculated using the TukeyHSD function in R.

**Table 2.** Summary of sex effect directionality across tissues.

	N metabolites higher in XX flies	N metabolites higher in XY flies
head	10	12
thorax	15	18
abdomen	11	13

Number of SD metabolites with higher levels in XX as compared to XY wildtype flies, or higher in XY as compared to XX wildtype flies, within the metabolome of each tissue.

Of all SD metabolites, only kynurenate maintained a significant sex difference in both wildtype and *dsx* null flies with elevated levels in XX flies of both SD groups across all three tissues (table 3). No other metabolites were significantly different between XX and XY flies in the *dsx* null SD group.

**Table 3.** Tissue-consistent SD metabolites.

head	effect size		adjusted *p*‐value (FDR)	
	wildtype	dsx null	wildtype	dsx null
1-METHYL-L-HISTIDINE	0.16	0.07	0.0003	0.3
DEOXYCARNITINE	−0.23	0.07	3.1x10^–5^	0.4
KYNURENATE	−0.52	−0.46	4.7x10^–9^	5.7x10^–8^
N-METHYLGLUTAMATE	0.19	0.15	0.01	0.2
ADMA	−0.22	−0.03	0.002	1
TYROSINE	−0.23	−0.01	0.05	1
VALINE	−0.24	−0.09	1.4x10^–5^	0.2

thorax	effect size		adjusted *p*‐value (FDR)	
	wildtype	dsx null	wildtype	dsx null
1-METHYL-L-HISTIDINE	0.15	0.03	9.5x10^–6^	0.7
DEOXYCARNITINE	−0.17	0.09	0.0001	0.2
KYNURENATE	−0.44	−0.44	5.3x10^–6^	2.3x10^–5^
N-METHYLGLUTAMATE	0.13	0.07	0.004	0.4
ADMA	−0.26	0.01	0.0003	1
TYROSINE	−0.12	−0.03	0.0002	0.9
VALINE	−0.24	−0.11	1.9x10^–5^	0.1

abdomen	effect size		adjusted *p*‐value (FDR)	
	wildtype	dsx null	wildtype	dsx null
1-METHYL-L-HISTIDINE	0.08	0.05	0.03	0.6
DEOXYCARNITINE	−0.17	0.13	0.01	0.2
KYNURENATE	−0.68	−0.56	2.1x10^–6^	4.1x10^–5^
N-METHYLGLUTAMATE	0.14	0.08	0.04	0.8
ADMA	−0.34	−0.06	0.0003	1
TYROSINE	−0.14	−0.05	0.004	0.9
VALINE	−0.17	−0.05	0.004	1.0

Effect size is calculated between XX and XY flies using TukeyHSD function in R. A positive effect size indicates values are higher in XY flies. ADMA refers to N,N-dimethyl-arginine.

### 2.2. *Dsx* influences sex differences in the metabolome in a tissue-specific manner

**Figure 3.**
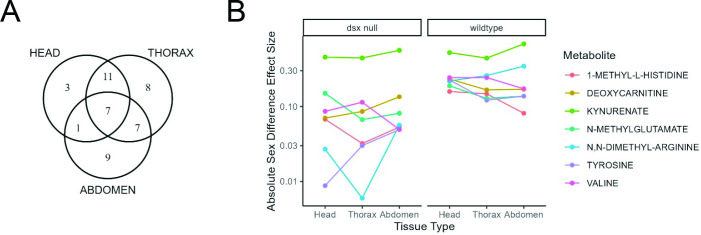
Tissue specificity of SD metabolites. (A) Venn diagram of number of SD metabolites that overlap across tissues. (B) Sex effect sizes for seven SD metabolites were significantly different between wildtype sexes across all three tissues. Effect sizes are calculated from the TukeyHSD function in R. Kynurenate, in light green, is the one metabolite that maintains a significant sex difference in both wildtype and *dsx* null flies across all three tissues. Effect sizes are plotted on a log10 scale.

Across tissues, the number and identity of SD metabolites varied (head = 22, thorax = 33, abdomen = 24), with thorax tissue sharing more SD metabolites with both the head and abdomen than the head and abdomen shared with each other (figure 3A). Each tissue also had unique SD metabolites, which were present but did not show SD at FDR < 5% in the other tissues. SD metabolites unique to the head included 3-nitro-L-tyrosine, gamma-aminobutyrate and nicotinamide mononucleotide. The thorax tissue had eight unique SD metabolites: agmatine sulfate, amino-isobutyrate, deoxy-guanosine, guanosine, histamine, pantothenate, phosphorylcholine (aka phosphocholine) and putrescine. Abdomen tissue had nine unique SD metabolites, including 4-imidazoleacetate, hippurate, hypoxanthine, kynurenine, L-carnitine, O-acetylcarnitine, ophthalmate, proline and spermidine. Seven metabolites were significantly different between wildtype sexes across all tissues (figure 3B). These features are 1-methyl-L-histidine, deoxycarnitine, kynurenate, N-methyl-glutamate, N,N-dimethyl-arginine, tyrosine and valine.

Overall, we found that the influence of *dsx* on SD in the metabolome is highly tissue specific. Some metabolites showed SD consistently across all tissues while SDs in other metabolites were unique to specific tissues. This tissue specificity could provide insights into how metabolic SDs contribute to the diverse physiological and behavioural traits observed between sexes.

### 2.3. SD metabolites are enriched in cellular growth and energy metabolism pathways

We next conducted pathway enrichment analysis for all SD metabolites and identified 11 pathways at FDR < 5% (electronic supplementary material, table S1C). The top five most significantly enriched pathways included phototransduction (KEGG ID: dme04745), branched-chain amino acid (BCAA) degradation (KEGG ID: dme00280), fatty acid elongation (KEGG ID: dme00062), dorso-ventral axis formation (KEGG ID: dme04320) and the mammalian target of rapamycin (mTOR) signalling pathway (KEGG ID: dme04150).

## 3. Discussion

The present study used a *Drosophila* model to investigate the potential for sexual dimorphism to impact SD in the metabolome. We measured 91 targeted metabolomic features across three tissue types, comparing wildtype with *dsx* null flies, which exhibit reduced sexual dimorphism. Our results suggest that metabolomic analysis can shed light on the ways in which fundamental genetic sex determination mechanisms shape downstream SDs at the molecular level.

The extent of *dsx*-dependent SD in the metabolome is a key finding of this study. Approximately half of the metabolites we measured showed significant SD in wildtype flies and SD in all but one metabolite was *dsx*-dependent. The *dsx* gene plays a pivotal role early in development in tipping the balance of gonad stem cells towards a ‘female’ or ‘male’ program [34], as well as directing sex-specific gene expression throughout adulthood, which influences anatomy and mating behaviours in both sexes [35–37]. In the absence of functional *dsx*, the gonad and genital discs of *Drosophila* develop a morphology that is intermediate to typically developing XX and XY flies [26]. By comparing the metabolome in this setting to the sexually differentiated wildtype, our results indicate that *dsx* influences not only anatomical differences that are clearly visible but also tissue-specific molecular differences that might, in turn, have important sex-specific physiological consequences.

One notable exception to the *dsx*-dependence in metabolome SD was kynurenate, a metabolite that consistently displayed higher levels in XX flies, regardless of *dsx*, suggesting that sex-specific patterns of kynurenate are *dsx*-independent. In our study, sex chromosome karyotypes were isogenic between SD groups, suggesting that the conserved SD in kynurenate is likely the result of an X- or Y-linked genetic factor. The gene(s) responsible for this is not known. However, two key genes at the top of the *Drosophila* tryptophan-kynurenine (Tryp-Kyn) degradation pathway, *vermilion* and *white*, are on the X chromosome [38,39], Therefore, metabolism of tryptophan and its derivatives, such as kynurenate, might have an X-linked sex-bias in *Drosophila*. More research is needed to test if the source of the SD in kynurenate is X-linked.

The tissue-specificity of wildtype SD was another critical finding of this study. Different tissues exhibited unique sets of SD metabolites that were mostly *dsx*-dependent. *dsx* modulates sex dimorphism in several ways. While the primary mechanism of *dsx* in early sexual development is through sex-specific splicing of *dsx*, giving rise to DSX^F^ and DSX^M^, which drive sex-specific transcription patterns, dimorphic expression of *dsx* in adults is controlled by sex-specific enhancers, which give rise to within-organ SD in *dsx* expression [28]. Furthermore, the sex-specific splice variants, DSX^F^ and DSX^M^, show sex-dependent activity on the expression of nearby genes [27]. The variability in sex effects on metabolite levels across tissues could thus reflect the within-tissue cell specificity of *dsx* expression, and/or the effect of DSX^F^ and DSX^M^ on downstream genes. That most SD metabolites in this study are *dsx*-dependent is a clear demonstration of the importance of sex-determination and sex differentiation on metabolism. Further work is needed to determine whether the influence of *dsx* on metabolite levels is a holdover from its expression earlier in development, or if *dsx* has a more direct effect on metabolic regulation within each tissue.

We observed that the thorax tissue, which houses much of the fat body, shared more SD metabolites with both head and abdomen than head and abdomen shared with each other. This could reflect the fat body’s importance to metabolism generally, and perhaps its role in inter-organ regulation of metabolism and developmental processes by hormonal signals [40]. Lazareva *et al.* [41] hypothesized that the fat body is the source of secreted circulating proteins that reach the brain via hemolymph to drive male-typical courtship behaviours, similar to *dsx*-regulated mechanisms underlying Yolk Protein [42] and Collagen IV [43] secretions. *Transformer* (*tra*), a sex development gene that regulates the sex-specific splicing of *dsx* pre-mRNA [44], also regulates sex-specific body fat levels [4,5]. It is unclear whether *dsx* plays a specific role downstream of *tra* in fat regulation. However, Clough *et al.* [27] identified 25 genes in the fat body that were differently expressed in response to a switch in DSX splice variants. Further experiments are needed to distinguish the roles of *tra*, *dsx* and downstream genes on metabolites in the fat body and other organs.

The SD metabolites we find enrich several biological pathways (electronic supplementary material, file S1), each of which may shed light on *dsx-*dependent SD. Among these pathways, we focus on the BCAA degradation and mTOR pathways. Our results reflect previous work on SD in the *Drosophila* transcriptome. Insulin-like signalling in flies acts through mTOR in the regulation of metabolism, growth and development, and has been a focus of work on SD in *Drosophila* [7,45,46]. Genetic inhibition of the insulin/mTOR axis has sex-specific effects on the head transcriptome, including female-specific reduction in the expression of genes in the BCAA pathway [47]. Here we show that SD in all three BCAAs (leucine, isoleucine and valine) were *dsx*-dependent in head and thorax tissues, which could be related to the sex-specific regulation of BCAA-pathway genes by insulin signalling. BCAAs are potent activators of mTOR signalling [48], and so the *dsx*-dependence of SD in BCAA metabolites we report suggests that *dsx* may lead to SD in growth and metabolism through BCAAs and, possibly, mTOR.

The mTOR pathway also mediates the longevity effects of dietary restriction [49], which has sex-specific outcomes in *Drosophila* [50]. In mice, restricting dietary BCAAs increased lifespan and metabolic health in males but not females [51]. This supports the idea that sex-specific regulation of nutrient metabolism, including BCAAs, contributes to the differential effects of dietary restriction observed between sexes. Taking this one step further, our work supports the idea that anatomical dimorphism could ultimately influence SD in the response to longevity interventions. Further research on the role of *dsx* in regulating BCAAs may provide insights into sex-related mechanisms that modify the response to *Drosophila* longevity interventions.

### 3.1. Limitations

This study has several limitations that should be considered. These experiments were designed to distinguish the effect of sex, defined by karyotype, in a mutant where the external morphology of the sexes is nearly indistinguishable [26]. To enable us to distinguish XX and XY flies without any clear external sexual dimorphism, we used a genetically marked Y-chromosome, Y-Bar[S], throughout the study. The bar-eyes phenotype manifests in eye morphology, which leads to a confound of eye morphology and karyotype. Thus, we acknowledge that some of the effects of sex on the metabolome, particularly in the head, may be associated with eye morphology. However, if this eye morphology were to explain the sex difference in the metabolome, there is no known way in which these effects would depend on the *dsx* mutation. Similarly, as is common in *Drosophila* studies involving P-element-induced alleles, like the *dsx* null, the presence of the mini-*white* gene in the P-element introduces a confound between the *dsx* genotype and the presence of mini-white. We note that *white* is a component of the Tryp-Kyn pathway, and so expression of mini-*white* might influence metabolism. However, of all metabolites, SDs in kynurenate were consistent across all tissues regardless of the presence of the P-element and so the mini-*white* genotype cannot explain these results. Last, the study examined head, thorax, and abdomen tissues, each of which includes multiple cell types. Thus, our tissue-specific approach cannot resolve effects at the cell-type level.

## 4. Conclusion

The findings from this study have several important implications for our understanding of variation in and regulation of physiological SD. First, they highlight the power of accounting for genetic mechanisms underlying sexual dimorphism, such as the role of the *doublesex* gene in *Drosophila*. The absence of *dsx* accompanies a significant reduction in SD in the metabolome, indicating that genetic context is crucial for understanding and interpreting SD in metabolic profiles. Second, the tissue-specific nature of *dsx* influence on the metabolome is consistent with prior studies showing that different tissues have distinct metabolomic profiles [15] and suggests that they are also differentially influenced by sex development gene networks. Comprehensive tissue-specific analyses can reveal insights into how SDs manifest in various organs. In conclusion, this study highlights the critical need for metabolomics research to incorporate genetic and phenotypic diversity related to sex characteristics.

## 5. Methods

### 5.1. Fly stocks and husbandry

We used two stocks containing *dsx* null deletions. Stock 1, *dsx*^f00683-d07058^ was obtained from the Bloomington *Drosophila* Stock Center with full genotype: w*; P+PBac{*w*[+mC] = XP3.WH3}*dsx*^f000683-d07058^/*TM6B***,** *Tb*^1^ [stock #66710]. To achieve isogenic lines, we first backcrossed this stock to our laboratory’s *w*^1118^ strain for five generations, tracking the *dsx* allele using the mini-*white* eye marker. As we did not observe viable homozygous *dsx* null offspring in this stock after crossing this line back to itself, we utilized a trans-heterozygous crossing scheme between our *dsx*^f000683-d07058^ strain and Stock 2, a *dsx* deletion stock gifted from the lab of Mark Siegal. This second stock, *dsx*^f01649-d09625^, had been previously crossed in the Siegal lab to a dominant-Bar[S]-marked Y chromosome in a w* background and carried an *ix* mutation balanced by *CyO* with full genotype: w*/B[S]Y*; ix*[GFP]/*CyO; Df(3R)f01649-d09625/TM6B, Tb*.

We crossed Stock 2 to our previously backcrossed Stock 1 and observed the expected proportions of *dsx* null flies relative to wildtype flies. For our metabolomics experiment, we discarded flies with straight wings to remove the *ix* mutation from our experimental samples and discarded flies with *TM6* to ensure that wildtype flies had an isogenic third chromosome. Thus, among the progeny of this cross, we recovered the four experimental genotypes (table 4).

**Table 4.** Experimental genotypes used in this study.

		genotype		
group	eye marker	chrom 1	chrom 2	chrom 3
XX – wildtype	white eyes	$\frac{w^{1118}}{w^{*}}$	$\frac{+}{CyO}$	$\frac{dsx^{f01649-d09625}}{+}$
XY – wildtype	white bar eyes	$\frac{w^{1118}}{B\left[S\right]Y}$	$\frac{+}{CyO}$	$\frac{dsx^{f01649-d09625}}{+}$
XX – dsx null	orange eyes	$\frac{w^{1118}}{w^{*}}$	$\frac{+}{CyO}$	$\frac{dsx^{f01649-d09625}}{dsx^{f00683-d07058}}$
XY – dsx null	orange bar eyes	$\frac{w^{1118}}{B\left[S\right]Y}$	$\frac{+}{CyO}$	$\frac{dsx^{f01649-d09625}}{dsx^{f00683-d07058}}$

### 5.2. Fly media and culture conditions

Flies were raised on a banana-based medium as described in [52] and housed in an incubator on a 12 h light-dark cycle at 25°C.

### 5.3. Tissue collection and metabolite extraction

All flies for metabolomics were collected 3 days post-eclosion and sorted with a two-minute timed exposure to CO_2_ into the four genotypes based on the segregating markers: orange eyes (mini-white), curly wings and bar eyes. Flies were then allowed to recover for 24 h in vials on fresh media before being flash-frozen in liquid nitrogen and stored at −80°C.

Individual flies were sectioned in a chilled petri dish on a cold metal block in dry ice, into head, thorax and abdomen samples using a chilled clean razor blade and sorted into 1.5 ml Eppendorf tubes for storage at −80°C. Each sample consisted of tissue from 10 flies, with four to five biological replicates per tissue per genotype.

For metabolite extraction, one 5 mm zirconium oxide bead was added to each Eppendorf tube before placing them in a frozen homogenizer block (Tissuelyser II, Qiagen). The tissue was pulverized by shaking at 30 Hz for 2 min in a cold room. Samples were then suspended in 1 ml of a methanol:H_2_O 4:1 solution kept on dry ice, after which each sample was vortexed for 10 s. Tubes were then centrifuged at 14,000 rpm for 15 min at 4°C. 600 µl of supernatant was transferred to a new 1.5 ml Eppendorf tube, dried under vacuum at 30°C overnight and stored at −80°C when retrieved the following morning.

### 5.4. Liquid chromatography-mass spectrometry (LC-MS)

Targeted LC-MS was carried out as described previously [18], providing measures of 91 metabolites.

### 5.5. Statistical methods

All statistical analysis was performed using R (version 4.3.0) open-source statistical software. Metabolite data were stratified by tissue (head, thorax, abdomen), then log-transformed, centred and scaled to have a mean = 0 and σ = 1 by sample. PCA was then performed using the ‘prcomp’ function in R.

### 5.6. Outlier detection

One replicate sample of XX-wildtype/abdomen (sample XXwA4) was reported by LC-MS Core staff as having aberrant values for several compounds and thus investigated as a potential outlier using the ‘boxplot.stats’ function in R. This function identifies outliers as values that fall beyond 1.5 times the interquartile range (IQR) from the first and third quartiles. Sample XXwA4 had 8 outlier metabolite values by this method, the most of all samples. We ran analyses both with and without this sample and found that when the sample was excluded, more metabolites were significantly different in abdomen between wildtype sexes at FDR<5% [30] vs 24). The complete 51 metabolome samples are included in our main analysis ([17]; 17 head, 17 thorax and 17 abdomen). Results of the abdomen analysis excluding sample XXwA4 are reported in electronic supplementary material, file S2.

### 5.7. Univariate analysis

To identify metabolites whose effect sizes differed between the four experimental genotypes in table 4, we first performed one-way ANOVA on each normalized metabolite within each tissue type (head, thorax, abdomen), using the ‘lm’ function in R, followed by post-hoc analysis using the ‘TukeyHSD’ function in R to retrieve effect sizes between XX and XY samples for each SD group. *p*-values from the TukeyHSD were adjusted for multiple comparisons within each tissue using the ‘p.adjust’ function in R with method = ‘fdr’. Metabolites that met an FDR cutoff of less than 5% were considered significantly different between the sexes in either wildtype, *dsx* null or both.

To determine whether there was a significant reduction in sex effect sizes across metabolites in each SD group, effect sizes between XX and XY from the Tukey HSD were compared between wildtype and *dsx* null by modelling the log-transformed absolute effect size as a function of the SD Group. *p*-values for these comparisons were derived from the ‘anova’ function in R on the linear models for each tissue.

### 5.8. Metabolite enrichment

Pathway analysis was conducted using the ‘FELLA’ package in R [53], which utilizes a network diffusion method to detect nodes in a biological network that are enriched for connectivity to small groups of metabolites, such as the 91 targeted metabolites measured in this study. Within FELLA, we accessed the *Drosophila melanogaster* KEGG Database (Release 111.0+). KEGG IDs were available for 85 of the 91 metabolites measured here. To test for enrichment of nodes within the KEGG network by a sub-set of the metabolites measured here, such as the metabolites with significant sex effects in our data, we permuted among the 85 metabolites 10,000 times to give empirical P values. We corrected for multiple testing by applying the ‘p.adjust’ function in R with method = ‘fdr’.

## Data Availability

Data and relevant code for this research work are stored in GitHub [54] and have been archived within the Zenodo repository [55]. Supplementary material is available online [56].
